# Serial Order Effect in Divergent Thinking in Five- to Six-Year-Olds: Individual Differences as Related to Executive Functions

**DOI:** 10.3390/jintelligence9020020

**Published:** 2021-04-02

**Authors:** Honghong Bai, Paul P. M. Leseman, Mirjam Moerbeek, Evelyn H. Kroesbergen, Hanna Mulder

**Affiliations:** 1 Department of Pedagogy and Education: Development & Education of Youth in Diverse Societies, Utrecht University, Heidelberglaan 1, 3584 CS Utrecht, The Netherlands; p.p.m.leseman@uu.nl (P.P.M.L.); h.mulder2@uu.nl (H.M.); 2 Department of Methodology and Statistics, Utrecht University, Padualaan 14, 3584 CH Utrecht, The Netherlands; m.moerbeek@uu.nl; 3 Behavioural Science Institute, Radboud University, P.O. Box 9104, 6500 HE Nijmegen, The Netherlands; e.kroesbergen@pwo.ru.nl

**Keywords:** divergent thinking, serial order effect, executive functions, selective attention, Alternative Uses Task, five- to six-year-olds

## Abstract

This study examined the unfolding in real time of original ideas during divergent thinking (DT) in five- to six-year-olds and related individual differences in DT to executive functions (EFs). The Alternative Uses Task was administered with verbal prompts that encouraged children to report on their thinking processes while generating uses for daily objects. In addition to coding the originality of each use, the domain-specific DT processes memory retrieval and mental operations were coded from children’s explanations. Six EF tasks were administered and combined into composites to measure working memory, shifting, inhibition, and selective attention. The results replicated findings of a previous study with the same children but at age four years: (1) there was a serial order effect of the originality of uses; and (2) the process mental operations predicted the originality of uses. Next, the results revealed that both domain-general EFs and domain-specific executive processes played a role in the real-time unfolding of original ideas during DT. Particularly, the DT process mental operations was positively related to the early generation of original ideas, while selective attention was negatively related to the later generation of original ideas. These findings deepen our understanding of how controlled executive processes operate during DT.

## 1. Introduction

Divergent thinking (DT) is a thinking process to generate original ideas by exploring many possible solutions to a particular problem (Guilford 1956; Wang et al. 2017). As an important component of creativity, DT has been the subject of abundant research. Tests of DT have been designed and related to other constructs, revealing individual differences and patterns of covariation with, for example, fluid and verbal intelligence (Silvia 2008), executive functions (Radel et al. 2015), and real-world creative achievement (Cramond et al. 2005; Runco et al. 2010), while also showing discriminant validity (Runco and Acar 2012). Still, DT as a process that unfolds in real time remains poorly understood, as are the relations of DT with general cognitive processes. This holds in particular with regard to the question whether DT is based on automatic, relatively effortless processes involving semantic and episodic memory (Brown 1973; Benedek et al. 2012b; Coney and Serna 1995; Mednick 1962; Milgram and Rabkin 1980), or also involves controlled, relatively effortful processes, pointing to possible involvement of executive functions (Beaty et al. 2016; Beaty and Silvia 2012; Beaty et al. 2014; Benedek and Fink 2019). The current study aims to address this issue by investigating the associations between individual differences in effortful processes and the real-time unfolding of original ideas in a DT task in young children.

A line of research informative to this question has focused on the so-called serial order effect. The serial order effect refers to the phenomenon that, while performing DT tasks (e.g., generating alternative uses of a brick in the Alternative Uses Task), participants often begin with generating many ideas in a short time frame, and these early ideas are usually relatively mundane and conventional. Gradually, participants tend to switch to generating more original ideas yet at a slower pace. The serial order effect has been reported in a range of age samples, from early childhood to adulthood (Bai et al. 2021; Christensen et al. 1957; Mednick 1962; Milgram and Rabkin 1980; Wang et al. 2017; Ward 1969). 

A possible explanation for the occurrence of the serial order effect is that DT involves activating automatic associations in memory (Benedek et al. 2012b; Brown 1973; Mednick 1962). Following spreading activation accounts of semantic and episodic long-term memory (Collins and Loftus 1975; Conway and Engle 1996), this process pertains to well-entrenched near connections first, therefore likely generating more conventional, familiar, or thematically related associations in memory. Activation will then only gradually spread to less well-entrenched, “weaker”, and more distal connections that are more likely to result in unconventional and truly novel alternative uses. The latter process presumably requires sustained effort and may be facilitated by domain-general intelligence (Beaty and Silvia 2012; Hass 2017). A related, but slightly different explanation for the occurrence of the serial order effect is that DT initially mainly involves generating alternative uses based on automatic associative retrieval from long-term memory, while more controlled, effortful thinking processes are called in when memory-based DT is becoming exhausted (i.e., the strongest connections have been activated and incorporated in the subject’s responses, and the core semantic network is fully exploited). This results in a gradual increase in qualitatively different thinking processes no longer based on finding (remote) memory associations, but on manipulating mental representations and creating new links between (parts of) representations, which may be facilitated by attention-based executive functions and working memory (Bai et al. 2021; Gilhooly et al. 2007). Both explanations suggest that originality in DT is promoted by controlled, effortful processing. Support for the involvement of controlled, effortful processing in divergent thinking and creativity is found in studies on adults (Beaty et al. 2014, 2016; Beaty and Silvia 2012; Benedek and Fink 2019). 

Even though one- and two-year-old children are already able to think divergently (Bijvoet-van den Berg and Hoicka 2014; Hoicka et al. 2016) and also the serial order effect has been confirmed to occur in childhood (Ward 1969), DT as a process that unfolds in real time is not well understood in children. To begin to unravel this issue, we previously conducted a detailed analysis of domain-specific processes occurring during a DT task, the Alternative Uses Task (AUT), in four-year-olds (Bai et al. 2021). In the AUT, children are instructed to generate as many unusual uses as possible for a number of stimuli (e.g., a picture of a brick). We found the serial order effect: later uses per object were more original. In addition to the standard procedure of the AUT, we asked children to explain how they came up with a particular use. Children referred to several categories of thinking processes in their explanations and two of them stood out. First, the process of retrieving uses from memory (e.g., “you can use a toothbrush to brush your teeth because I do it every day”; similar to memory-based production in a study on adults by Gilhooly et al. 2007) supported the generation of the largest number of uses compared to other processes. Second, the process of performing mental operations on the stimuli (e.g., “you can take the hairs [of a toothbrush] off and use them as eyelashes of a doll”; similar to disassembly-use production in Gilhooly et al. 2007) was found to uniquely predict the originality of uses. 

Notably, these thinking processes also showed serial order effects, although in different directions. Memory retrieval occurred frequently initially, but the occurrence decreased with the increasing rank of a use in the sequence of all mentioned uses for a particular stimulus. The process mental operations occurred very infrequently initially, but the occurrence increased with increasing rank. Visual inspection of the graphs representing the serial order effects of originality and the process mental operations revealed co-occurrence of the rise, peak, and levelling-off, suggesting that especially this process may underlie relatively original responses (Bai et al. 2021). We argued that, in contrast to memory retrieval based on automatically activated memory associations, the process of performing mental operations on a stimulus is executive and effortful in nature, given that performing mental operations on a stimulus involves holding an image of the object in mind while manipulating it, inhibiting the perceptual or representational salience of the object in its usual whole integrated form, and shifting attentional focus from global, holistic to local, singled-out features of the stimulus in order to operate on them. 

To date, several studies have investigated the association between DT performance, such as originality, and domain-general executive function skills. As an umbrella term, executive functions (EFs) refer to the goal-oriented regulation of one’s thoughts, actions, and emotions (Moriguchi et al. 2016), involving a set of top-down, effortful mental processes. There is general agreement that there are three core EFs (Diamond 2013; Miyake et al. 2000): (1) inhibition, which refers to the ability to suppress one’s predominant behavioral tendencies, thoughts, and/or emotions, in favor of sub-dominant responses; (2) working memory, which refers to the ability and capacity to hold information in mind while mentally processing that information; and (3) shifting, which refers to the ability to adapt one’s thoughts and behaviors to the changing needs of a task or situation. 

Study findings regarding the association between working memory, shifting and DT are rather consistent, typically showing that with enhanced working memory and shifting ability, participants performed better on DT tasks (de Dreu et al. 2012; Kharkhurin and Wei 2015; Lee and Therriault 2013; Lu et al. 2017; Nusbaum and Silvia 2011; Yeh et al. 2015; Zabelina et al. 2019; Zabelina and Robinson 2010). Possible explanations are that a relatively large working memory capacity enables participants (1) to hold more information in mind so that complex, uncommon associations can be formed (Zabelina et al. 2019) and (2) to simultaneously hold in mind and evaluate several candidate responses to select only original ideas (Lee and Therriault 2013). In addition, the ability to flexibly shift between the analytic, top-down and the associative, bottom-up thinking modes (Pringle and Sowden 2017; Zabelina and Robinson 2010) or between different task stimuli (Lu et al. 2017) may help participants to generate out-of-the-box responses. 

However, findings regarding the associations between inhibition and DT are mixed, with some studies showing a positive association (Benedek et al. 2012a, 2014; Edl et al. 2014; Krumm et al. 2018; Rominger et al. 2017; Zabelina and Ganis 2018) and other studies showing a negative association (Radel et al. 2015; Scibinetti et al. 2011), or no association (Stolte et al. 2019; Zabelina et al. 2019). Some scholars have argued that enhanced inhibition ability is positively related to DT performance, because inhibition is beneficial for suppressing common and already-generated ideas while staying focused on the task and ignoring distraction (Beaty and Silvia 2012; Beaty et al. 2014; Benedek et al. 2012a, 2014). In contrast, others have proposed that reduced inhibition is conducive to DT by allowing information that is only peripherally related to the stimulus to enter working memory (Radel et al. 2015), thus broadening the scope of attention and enhancing the breadth of input for further processing (Stolte et al. 2019; Zabelina 2018). 

Only a few studies to date, involving adult samples, have investigated the relationships between domain-general EFs and the serial order effect of originality in DT, showing inconsistent findings. In one study, Wang et al. (2017) investigated how inhibition, working memory, and shifting moderated the serial order effect of the originality of uses in the AUT. Shifting, but not inhibition or working memory, was found to significantly moderate the serial order effect. Adults with higher shifting ability performed better throughout the task and showed the serial order effect (i.e., originality of uses was higher later on in the series of mentioned uses), whereas adults with lower shifting ability generated more mundane ideas overall and did not show the serial order effect. Further, one study reported a reduced serial order effect in individuals with high EF (Beaty and Silvia 2012), while another study showed no moderation of the serial order effect by EF (Hass 2017). To summarize, there is some evidence that EF and DT are related, but results to date are inconsistent, and it is currently unclear how EFs affect the process of idea generation during DT tasks.

### The Present Study

The present study is partly a replication of our previous study using the same sample of children but now at the age of five to six years, about one and a half years later than in our previous study. Furthermore, the present study extends our previous work by examining the role of EFs in DT through relating individual differences in domain-general EFs to the process of DT, in terms of both the serial order effect in originality and the domain-specific processes of memory retrieval and mental operations. Studying the association of DT and EFs in young children is relevant for two reasons: (1) Research has suggested that formal schooling reduces DT because of the strong focus on convergent thinking in teaching (Gralewski et al. 2016; Krampen 2012; Russ and Wallace 2013). Therefore, it is important to know if and how EFs affect DT at an early age, before formal schooling begins. (2) Young children demonstrate similar involvement of domain-specific effortful DT processes in novel idea generation during a DT task as adults (Bai et al. 2021; Gilhooly et al. 2007). However, to the best of our knowledge, no study to date has examined the relationship between EF and domain-specific DT processes in children this young.

Our specific research aims were as follows. First, we aimed to investigate whether the serial order effect of originality as reported in four-year-olds in our previous study (Bai et al. 2021), was also present in five- to six-year-olds. If replicated, this would strengthen the evidence base that the serial order effect—as a well-known effect occurring in DT in older children and adults—is already present in very young children. Second, we aimed to test if the two most important DT processes that we identified in our previous study in four-year-olds, i.e., mental operations and memory retrieval (Bai et al. 2021), would also occur in five- to six-year-olds and would be related to originality in a similar way at this age. Specifically, we expected that memory retrieval would occur relatively frequently overall but would not relate to originality, whereas mental operations would occur much less frequently overall but would relate positively to originality. 

Third, in addition to testing whether we could replicate findings from our previous study, we aimed to expand on the previous study by exploring how domain-general EFs and domain-specific processes play a role in the unfolding of original ideas. To this end, we systematically investigated the interactions between domain-general EFs, domain-specific DT processes (memory retrieval and mental operations), and use rank in predicting the originality of uses. We formulated two hypotheses regarding the moderating effects. First, we expected the process of mental operations to become increasingly important as a predictor of the originality of a generated use the later in the sequence of all uses mentioned for a particular stimulus object. This hypothesis was based on the findings in our previous study (Bai et al. 2021), suggesting a gradual shift from responses based on associative memory processes (comparatively less original) to responses based on controlled mental operations (comparatively original). Second, we expected the effect of individual differences in EFs, including selective attention, on originality to increase with use rank based on the theoretical consideration that a gradual shift from automatic associative responses to controlled executive processing would demand stronger involvement of domain-general EF capacities and, hence, would enlarge the effect of individual differences therein. Relatedly, we expected an interaction effect of EFs and the DT process mental operations on originality, presupposing that reliance on domain-specific mental operations in generating novel uses for a particular object, would be facilitated by a higher level of domain-general EF abilities (and impeded by a lower level in EFs). 

## 2. Materials and Methods 

### 2.1. Participants

The present study is part of a longitudinal research project which investigates the development of divergent thinking in young children. The project was approved by the Ethics Review Board of the Faculty of Social and Behavioural Sciences of Utrecht University in 2016 (reference number: FETC16-066). One hundred and seven children (49 boys and 58 girls; age at the first measurement ranged from 3.87 to 5.10 years, *M* = 4.44, *SD* = 0.26) with informed, active parental approval from four typical primary schools in the Netherlands participated in this research project. The longitudinal project followed children for about one and a half years, from the age of four years. Divergent thinking was measured every six months at age four, four to five, five, and five to six years. The current study used data from the last measurement wave, in which children were also given a battery of domain-general EF tasks. 

### 2.2. Measures

#### 2.2.1. Divergent Thinking

To assess children’s DT, we used the Alternative Uses Task (AUT; Guilford 1967). Children were first instructed that they would see a number of images of common objects and that, for each object, they should think of as many different and unusual uses as possible. Subsequently, a real newspaper was presented to children and used as an example object. The experimenter first asked children what a newspaper could be used for to start the conversation, and then gave several unusual uses of the newspaper and explained how she came up with these uses (e.g., “you can fold the newspaper, then you have a hat”), and eventually encouraged children to give unusual uses and explain their thinking. The purpose of this example was to ensure that children understood the task. Next, the formal testing began and six pictures of common objects, including a lunch box, a tire, an umbrella, a pencil, a shovel, and a toothbrush, were presented to children one by one. Note that in the longitudinal study that the current study is part of, we used two equivalent stimulus sets for the AUT alternately at successive measurement waves. The previous study (Bai et al. 2021) used data from wave 1 in which set-1 stimuli were used. The current study used data from wave 4 in which set-2 stimuli were used. Equivalence of the stimulus sets was confirmed in another study (Bai et al. under review), where the variable stimulus set had no significant effect on AUT performance across different measurement waves. For each object, children were asked to generate as many different and unusual uses as possible and to explain their thinking process in relation to the generation of these uses. To encourage children to generate more uses and to report on their thinking process, the task was conducted with interactive dialogues (van Someren et al. 1994). Children were prompted to think of more uses (e.g., “What else can you use a basket for?”), to elaborate on a generated use (e.g., “How do you do that?”, “Can you tell me more about it”), and to explain how they came up with a use (e.g., “How did you come up with this idea?”, “Have you done it before?”). The experimenters were instructed to find a balance between prompting children after *each* generated idea, without however breaking the thinking flow of children or making them feel uncomfortable with the test. Note that children sometimes also gave explanations spontaneously and did not need to be prompted for those particular uses. All children were prompted, while children who were less expressive or whose utterances were more idiosyncratic and hard to grasp, received more prompts. No strict time limit was set for the test considering that children might differ from each other regarding the time needed to fully express their thinking. The test took about 15 to 35 minutes, including the instructions and necessary breaks. All test sessions were video-recorded for later coding. 

**Assessing DT ability.** Although it is common to derive several DT ability measures from tasks such as the AUT (e.g., fluency, flexibility, originality; see Runco and Acar 2012), we focused specifically on originality in the present study, as originality is generally considered the core feature of divergent and creative thinking (Runco and Jaeger 2012; Smith and Smith 2017; Simonton 2012; Weisberg 2015), while fluency and flexibility are regarded as facilitating originality (Nijstad et al. 2010; Silvia et al. 2008; Forthmann et al. 2018). Following past research (van de Kamp et al. 2016; Krampen 2012), we first coded all mentioned uses per object based on the type of actions implicated in these uses. For instance, using a brick “to build a house” and “to build a bridge” were coded as the same type of action, i.e., “to build something” for the object brick (see Appendix A for a list of action types per object). Subsequently, we scored the originality of the action types, per object, based on how often that action type was mentioned in the whole sample, using the following equation: Originality of an action type = 1 − (The number of participants who mentioned one or more uses that implicated this action type/The total number of participants). Eventually, all uses received a score for originality based on the originality of the implicated action type. Note that the originality of uses had a nested data structure: the originality scores of the uses were nested in objects and the objects were nested in children. These data were used in the main analyses. 

In addition, we also derived child-level fluency and originality scores for descriptive purposes. Fluency referred to the average number of uses children generated across objects. Originality was calculated following two steps: first the originality scores of action types per object were summed, then the sums per object were averaged per child.

**Domain-specific DT processes.** All video recordings of the AUT test sessions were verbatim transcribed. Content relevant information, including children’s use of signs and gestures, objects in the test environment that children referred to, whether the test was interrupted, and so on, were noted in the transcripts to facilitate correct understanding of children’s utterances. The transcripts were then divided into episodes, with each episode covering a coherent stretch of dialogue that was related to a single generated use. Subsequently, the coding scheme of Bai et al. (2021) was applied by the first author to assess the processes of DT. For the purpose of the present study, we focused on the two key domain-specific DT processes as identified in the previous study, namely memory retrieval, which was found to be most frequently referred to by children, and mental operations, which was found to uniquely predict the originality of uses (Bai et al. 2021). For each episode, we determined whether children, in explaining their thinking, referred to memory retrieval (scored 1) or not (scored 0) and to mental operations (scored 1) or not (scored 0). Note that within a particular episode, children could refer to none, one, or both DT-processes. Table 1 presents the definitions and a number of examples of these processes. To establish inter-coder reliability, 15% of the transcripts were double-coded by a trained second coder (a master’s student in educational sciences). Cohen’s kappa’s showed moderate to substantial agreement between the two coders, κ = .80 for memory retrieval and κ = .69 for mental operations (Landis and Koch 1977; see Appendix B for an overview of the inter-coder reliability). Similar to originality, the data of the DT processes also had a nested structure: the occurrence of a DT process per mentioned use was nested in objects and the objects were nested in children. These data were used in the main analyses.

In addition, we also calculated child-level scores of the DT processes for descriptive purposes. Child-level DT process scores were computed as the average frequencies of occurrence of the processes memory retrieval and mental operations in the generation of uses across objects.

#### 2.2.2. Executive Functions

Several measures were used to assess domain-general EFs. In addition to tests of inhibition, shifting, and working memory, we included a test of selective sustained attention as a general component of EF which is especially relevant at younger ages (Hendry et al. 2016; Veer et al. 2017). 

**Inhibition.** Two tasks were used and programmed in E-prime (Psychological Software Tools, http://www.pstnet.com, accessed on 31 October-2020) to assess inhibition. The *Go/NoGo* task measures the extent to which children can inhibit a dominant motor response (Newman et al. 1985). The test is reported to have satisfactory psychometric quality, with a test–retest reliability of *r* = .65 (Nguyen et al. 2018). Children were instructed to press the space key when “go” stimuli (pictures of ten common objects, e.g., a book) were shown on the screen, and not to press the space key when the “no go” stimulus was shown (a picture of a dog). After introduction to the task and a practice session, children performed the test session in which the go and no go stimuli were shown 40 and 20 times, respectively. Each stimulus was presented for 1000 ms, and the intervals between two stimuli varied from 200 ms to 1200 ms so that children were not able to predict the appearance of a stimulus. The order of stimulus presentation was pre-randomized and the same for all participants. The frequency at which children pressed the space key when they were shown the no go stimulus, and thus failed to inhibit their dominant motor response, was calculated. 

The *Animal Stroop* task measures the extent to which children can inhibit their reaction towards a salient feature and name a sub-dominant feature of a stimulus (van der Ven et al. 2012). The test-retest correlations of three separate measures derived from this task over an 18-month interval in a sample of children aged six years at the first measurement occasion are reported to range between *r* = .28 and *r* = .33, and using confirmatory factor analysis all measures were found to load significantly on a combined inhibition-shifting factor (van der Ven et al. 2012). In this task, children were first given the control block in which typical animal drawings of a cow, sheep, duck, and pig were presented (head–body congruent stimuli). Children were instructed to give the name of the animal as soon as possible when a drawing appeared on the screen. In this block, each animal was presented 12 times, yielding a total of 48 trials. Next, children were given the test block in which a mix of drawings was presented: some drawings showed an animal body with a human head (control stimuli), while others showed an animal body with the head of another animal (head–body incongruent stimuli). Again, children were instructed to give the name of the animal for the body part (thus not for the more salient head part) as soon as possible each time a drawing appeared on the screen. There were 12 drawings for both the control and the head–body incongruent stimuli, and each drawing was presented four times, yielding a total of 96 trials. Presentation of each stimulus was preceded by a red fixation cross shown for 400 ms. The order of stimulus presentation was pre-randomized and the same for all participants. Instructions and practice trials were given at the beginning of each block (control and test). During the practice trials, the experimenter gave feedback and corrected children’s responses when they named the wrong animal (e.g., “Very good”, or “Oops, that was a pig”); while during the test trials, no feedback was given. The percentage of head–body incongruent trials in which children correctly named the animal name of the body part was calculated, following the recommendation by van der Ven et al. (2012). 

To strengthen the measurement quality, composite inhibition scores were computed by averaging the *z*-standardized scores of the Go/NoGo (reversed coded) and the Animal Stroop tasks (the correlation between the two measures as included in the composite is *r* = .21). The composite scores were used for further analysis. 

**Shifting.** Two tasks were used to assess shifting. The *Dimensional Change Card Sort* (DCCS) measures the extent to which children can shift to a new rule and abandon a previously correct rule. The DCCS has been widely applied to assess young children’s cognitive flexibility, also referred to as shifting ability (Doebel and Zelazo 2015). The test–retest reliabilities of different versions of the DCCS are reported to range between *r* = .69 and *r* = .90 (Beck et al. 2011). An advanced version of the DCCS for five-year-olds was used in the present study, which included three blocks (Beck et al. 2011; Zelazo 2006). During the test, two boxes with a slot on the top-side were placed next to each other in front of children, with one box labelled with a card that showed a blue star and the other box labelled with a card that showed a red truck. In the first block, children were instructed to sort six cards by color (“color game”), of which three showed a red star and three a blue truck. Therefore, the cards with a red star should go into the slot of the red-truck box, while the cards with a blue truck should go into the slot of the blue-star box. In the second block, children were instructed to sort a new set of the same cards by shape (“shape game”). Other than the color game, the cards with a red star should now go into the blue-star box, and the cards with a blue truck should go into the red-truck box. In the third block, children were instructed to sort again a new set of twelve cards, of which six cards were identical to the cards used in the first and second blocks, and another six cards had identical figures (three red-star cards and three blue-truck cards) but now with a black border. The sorting rule was more complex in this block: if a card had a black border, the card needed to be sorted by color; if a card did not have a black border, the card needed to be sorted by shape (“advanced sort”). Every time a card was presented to the children, a rule reminder was given by the experimenter. The order of card presentation was pre-randomized and the same for all participants. Instructions were given at the beginning of each session. Additionally, following the instructions, for the color game, two practice trials were given; and for the shape game and the advanced block, the experimenter confirmed whether the children still remembered the sorting rules, for instance by asking “Can you show me where the stars go in the shape game?” and “What game do you play if there is a border?” Children’s shifting scores were determined as follows: A score of zero was given when children sorted less than five out of six cards correctly in the first block and thus failed on color sort; A score of 1 was given when children succeeded on color sort, but sorted less than five out of six cards correctly in the second block, and thus failed on shape sort; A score of 2 was given when children succeeded on both color and shape sort, but sorted less than 9 out of 12 cards correctly in the third block; Finally, a score of 3 was given when children succeeded on both color and shape sort and sorted at least 9 out of 12 cards correctly in the third block.

The *Animal Shifting* task measures the extent to which children can shift between applying different rules (van der Ven et al. 2012). The test–retest correlations of measures derived from this task over an 18 month-interval in a sample of children aged six years at the first measurement occasion are reported to be between *r* = .32 and *r* = .37, and all derived measures loaded significantly on a combined inhibition-shifting latent factor (van der Ven et al. 2012). The task was programmed in E-prime. Children were first given a control block and then a shifting block, and each block consisted of 40 trials. In the control block, for each trial, a picture of an animal (cat, dog, bird, or fish) or a fruit (strawberry, pear, cherry, or banana) was shown centrally on the screen. Children were instructed to name the animal or fruit as quickly as possible. In the shifting block, for each trial, a picture of an animal and of a fruit were shown simultaneously (one on the left side and one on the right side of the screen). Children were instructed to name either the animal or the fruit as quickly as possible. If the background color of the screen was yellow, children should name the fruit, and if the background color was purple, children should name the animal. Presentation of each stimulus was preceded by a red fixation cross shown for 700 ms. The order of stimulus presentation was pre-randomized and the same for all participants. Instructions and practice trials were given at the beginning of each block (control and shifting). During the practice trials, the experimenter gave feedback and corrected children’s responses when they named the wrong animal or fruit (e.g., “Very good”, “Oops, that is not right. It was a pear”). Additionally, for the shifting practice trials, a rule reminder was given when children gave incorrect responses. No feedback was given during the test trials. The percentage of trials in which children correctly named the animal or fruit in the shifting block was computed as a measure of shifting ability, following the recommendation of van der Ven et al. (2012).

To strengthen the measurement quality, composite shifting scores were computed by averaging the *z*-standardized scores of the DCCS and the Animal Shifting tasks for each child (the correlation between the two tasks is *r* = .21). These composite scores were used for further analysis. 

**Working Memory.** To measure children’s working memory, a computerized verbal *Word Recall Backwards* task as adapted by Verhagen and Mulder (2013) was used. The task was programmed in E-prime. Children listened to series of two and three monosyllabic words voiced by a laptop computer, and were instructed to recall the words out loud in the reversed order. After the instruction and a short practice session, children continued with the test, which included six trials: three two-word trials and three three-word trials. The order of presentation of the trials was the same for all participants. The percentage of trials for which children correctly recalled the words in the reversed order was calculated as a working memory score and used in the analyses. 

**Selective Attention.** To measure selective attention, a computerized visual search task programmed in E-prime for children aged two to five years was used (Mulder et al. 2014; Veer et al. 2017). In Veer et al. (2017), the test–retest correlation of this task administered at 2.5 and 3 years using an age-appropriate version for toddlers was *r* = .55. In two studies, the selective attention measure was found to be significantly associated with other executive function measures, in particular spatial working memory, verbal short term memory, and inhibition (Mulder et al. 2014; Veer et al. 2017). In the present study, the version for five-year-olds was used. In this test, children had to find and point to targets (elephants) as quickly as possible while ignoring distractors (bears and horses of similar size and color as the targets). Four test trials were given, presenting a grid-structured screen with targets and distractors. Each trial was shown for 40 s. The target to distractor ratio increased from 1:5 for the first two trials (presented on a 6 × 8 grid screen) to 1:8 for the third trial (presented on a 9 × 8 grid screen), and 1:24.5 for the fourth trial (presented on a 17 × 12 grid screen; the animal images in this display were smaller compared to previous trials to fit the screen). Instructions and practice trials were given at the beginning of the task. After finishing each test trial, verbal and visual rewards were given (e.g., a picture of an elephant holding a flower was shown as a brief break in between test trials; the experimenter would say “Well done!”, “Good job!”). Additional instruction and two extra practice trials were given before the fourth test trial in order to familiarize children with the smaller images. Children were continuously prompted to keep searching and finding as many elephants as quickly as possible on each trial. Each time children pointed to a target, the experimenter would press a key so that the target would be crossed off by a diagonal blue line on the screen. The average number of targets children identified across all test trials was calculated and used as a measure of selective attention in the analyses. 

### 2.3. Procedures

All children were tested individually in a separate room at their schools. Research assistants, students in educational sciences, were trained to administer the tests. They read the test instruction manual carefully, watched videos of example test sessions, conducted and videotaped a pilot test, and received extensive feedback. In addition, the first author supervised the first two to three test sessions of each assistant. 

### 2.4. Data Analyses

#### 2.4.1. Missing Data and the Final Sample

The current study included 102 out of 107 children of the first measurement wave of the longitudinal project (48 boys and 54 girls; age ranged from 5.45 to 6.53 years, *M* = 5.93, *SD* = 0.27). Four children had moved to other schools, and one child was very shy and refused to participate in any test. Appendix C shows an overview of the missing data and reasons for missingness across the different tasks. 

#### 2.4.2. Statistical Analysis

We investigated whether use rank (the rank number of a use in the sequence of all mentioned uses for a particular stimulus object), the occurrence of the domain-specific DT processes of memory retrieval and mental operations, domain-general EFs, and also the interactions between these variables predicted use originality. As the outcome variable use originality had a three-level nested data structure (i.e., level 1: use, level 2: object, level 3: child) and constituted proportional data, multilevel logistic regression analyses with a binomial distribution were run. As a first step, we tested the serial order effect of originality by building an intercept-only model (Model 1; M1) and a model with the linear and quadratic effects of use rank (M2). As a second step, we examined whether the DT processes predicted the originality of uses. We built separate models to test the main effects of memory retrieval and mental operations (M3), considering that the power of the analysis might be limited because (1) the sample size of our study was rather small (102 children) and (2) the distributions of both DT processes were skewed. In particular, the DT process mental operations occurred rather infrequently. Next, to explore the role of domain-general EFs in DT, we added progressively the main effects of the EFs and several within-level and cross-level interaction effects to the model. Specifically, the following models were run, separately for the DT processes memory retrieval and mental operations: a model with the within-level interaction effect of DT process by use rank (M4); and for each EF separately, a model with the main effect of the EF concerned and the cross-level interaction effects of use rank by EF and DT process by EF, allowing the effects of use rank and DT process to vary across children (M5). The multilevel models were fitted in SuperMix version 2.1 (Hedeker et al. 2008), using adaptive quadrature estimation with 20 quadrature points. All predictors were grand-mean centered before running the analyses to avoid multicollinearity. For interpreting the results, the *p* < .05 level of significance was applied. 

## 3. Results

### 3.1. Descriptive Statistics

Descriptive statistics for the EF tasks and the aggregated child-level measures of the AUT are shown in Table 2. Children generated on average 4.21 uses per object on the AUT. For about 43% of the uses, children gave an explanation of their thinking processes by referring to memory retrieval, for 12% of the uses the explanation referred to mental operations, and for 5% of the uses children’s explanations referred to both memory retrieval and mental operations. For the remaining uses (about 51%) either no explanation was provided or an explanation that could not be coded as one of the two DT processes. Table 3 shows the correlations between the EF measures and the child-level measures of the AUT, and Table 4 presents the number of uses by use rank regarding children’s references to the DT processes memory retrieval and mental operations. Note that the vast majority of children (99/101; 98%) referred to memory retrieval at least once for explaining their uses, and 75 children (74%) referred to mental operations at least once (see Table 4). 

### 3.2. Multilevel Regressions 

The results of multilevel regressions are presented in Table 5 (models M1–M4) and Table 6 (models M5). 

#### 3.2.1. Serial Order Effect of Originality

**Figure 1:**
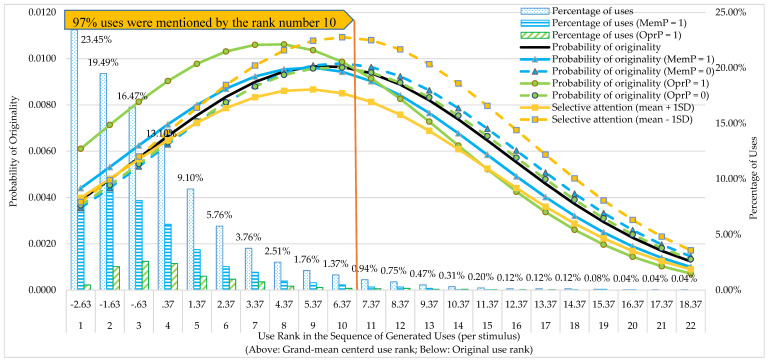
The probability of the originality of uses by use rank under different conditions. MemP = memory retrieval and OprP = mental operations. For a better visual presentation, we plotted this figure based on estimates of an additional full model (*N* = 100) which included the linear and quadratic effects of use rank (coefficients were respectively 0.15 and −0.013), the main effects of memory retrieval, mental operation, and selective attention (coefficients were respectively 0.14, 0.37, 0.020), and interaction effects of use rank by memory retrieval, use rank by mental operations, and use rank by selective attention (coefficients were respectively −0.028, −0.054, and −0.016). The estimations of coefficients were very close to results presented in Table 5 and Table 6 (difference ranges from 0.001 to 0.01).

First, we tested whether the serial order effect was present in the originality of uses. Compared to the intercept-only model (M1), adding the linear and quadratic effects of use rank (M2) significantly improved the model fit, as indicated by the change in model deviance: Δ_deviance_ (2) = 145.01, *p* < .001. Both linear and quadratic effects of use rank were significant. The positive coefficient of the linear effect indicated that there was a serial order effect of originality, meaning that the later a use was generated in the sequence of all mentioned uses per object, the higher the probability that this use was scored as original. Conversely, the negative coefficient of the quadratic effect of use rank indicated that the increasing probability of uses being scored as original levelled off and even started to decrease towards the end of idea generation (see the black curve in Figure 1).

#### 3.2.2. DT Processes and Originality

Next, we investigated whether memory retrieval and mental operations predicted the logit probability of the originality of uses in separate models (i.e., model M3–MR and M3-MO in Table 5, respectively). Adding the main effects of DT processes significantly improved the model fit compared to M2: for memory retrieval, Δ_deviance_ (1) = 5.82, *p* < .05; for mental operations, Δ_deviance_ (1) = 20.97, *p* < .001. The results show that, although both memory retrieval and mental operations significantly and positively predicted the logit probability of the originality of uses, the effect of mental operations was somewhat larger than the effect of memory retrieval (for memory retrieval: odds ratio [*OR*] = 1.13; for mental operations: *OR* = 1.38).

#### 3.2.3. Full Models with EFs for Memory Retrieval

Finally, we investigated how domain-general EFs, domain-specific DT processes, and use rank interacted in predicting the logit probability of the originality of uses. For the DT process memory retrieval, adding the within-level interaction of memory retrieval by use rank (see model M4–MR in Table 5) only marginally improved the model fit (*p* < .10), and the coefficient of the interaction was only borderline significant (*p* = .07). In Figure 1, we plotted the probability of originality by use rank for both conditions by whether references were made versus not made to the process memory retrieval. As shown in the Figure, the serial order effect of originality did not change much dependent on whether references were made to memory retrieval (the blue solid curve) or not (the blue dashed curve). Additionally, as shown in Table 6, in the full models (M5) which included also the main effect of EF and the cross-level interactions of use rank by EF and memory retrieval by EF, with each EF construct tested in a separate model, only the interaction of selective attention by use rank was borderline significant (*p* = .052). Note that, due to missing data on the EF measures, the number of participants included in the M5 models differed from the M4 models. As a consequence, a direct comparison of model deviances of M4 and M5 models for each EF was not possible. Therefore, the change in model deviance is not reported here.

#### 3.2.4. Full Models with EFs for Mental Operations

For the DT process mental operations, adding the within-level interaction of mental operations by use rank significantly improved the model fit, and the coefficient of the interaction was also significant (see model M4–MO in Table 5). This interaction effect is also plotted in Figure 1. As shown in the Figure, for uses generated before the rank number of 10, which comprises about 97% of all mentioned uses, uses that were explained with references to the process of performing mental operations on the stimulus (the green solid curve) were evidently more original than uses that were explained without such references (the green dashed curve). For uses generated after the rank number of 10, which took up only 3% of all mentioned uses, an opposite pattern can be observed: uses that were explained with references to the process mental operations were less original than uses that were generated without such references. Next, largely in line with the findings for the process memory retrieval, in the full models that included the main effect of EF and the cross-level interactions of use rank by EF and mental operations by EF (M5 models in Table 6), only the interaction of selective attention by use rank was found to be significant. In Figure 1 we also plotted the probability of originality by use rank for high (the orange solid curve; one *SD* above the mean) versus low selective attention (the orange dashed line; one *SD* below the mean). Note that the *SD* and mean were calculated based on the disaggregated data of selective attention at the use level. As the figure shows, before use rank three, there is no observable difference in the probability of originality of uses between both levels of selective attention, while beyond this rank children with lower selective attention generate consistently more original ideas. In addition, the difference between the levels of selective attention first increases, until approximately use rank 12, and then decreases gradually. 

## 4. Discussion

The goal of the present study was twofold. First, we aimed to replicate the findings of our previous study on DT in four-year-olds (Bai et al. 2021). Specifically, we investigated whether the serial order effect of originality would occur again in the same group of children, but now at the age of five to six years. In addition, we investigated whether the two domain-specific DT processes, retrieval from episodic and semantic long-term memory and performing mental operations on the stimulus, predicted the originality of the generated ideas. It should be noted that we used a different stimulus set for the AUT than in the previous study (Bai et al. 2021). The equivalence of the stimulus sets was confirmed in another study (Bai et al. under review). Despite the difference in stimulus sets, the results largely confirmed our expectations: (1) There was a serial order effect regarding the originality of mentioned uses within the AUT, that is, uses generated later in the sequence of all mentioned uses of a particular stimulus object were more original, at least until, roughly, rank 10 in the sequence of mentioned uses (after 97% of all uses were mentioned), as Figure 1 reveals; (2) The DT processes memory retrieval and mental operations both positively predicted the overall originality of uses, while mental operations showed a stronger effect on originality than memory retrieval.

Second, the present study also aimed to deepen our understanding of how controlled executive processes operate during DT and how they may moderate the serial order effect of originality. We systematically investigated the interactions between domain-general EFs, domain-specific DT processes, and use rank, regarded as a quasi-time variable, in predicting the originality of uses. The findings can be summarized as follows. First, there was an interaction effect of the DT process mental operations and use rank in predicting the originality of uses. Second, selective attention, but none of the other EFs examined in this study (shifting, inhibition, and working memory) moderated the serial order effect of originality. Finally, there was no interaction effect of domain-general EFs and domain-specific DT processes on originality. Next, we discuss each of these findings in turn.

The serial order effect of originality in DT is already well-established in older children and adults (Christensen et al. 1957; Mednick 1962; Milgram and Rabkin 1980; Wang et al. 2017; Ward 1969). The present study among five- to six-year-old children replicates our previous study with the same children when they were four years of age (Bai et al. 2021), and both studies add to the body of evidence by confirming the serial order effect in young children. The serial order effects at different ages revealed a similar increase of originality by use rank. There were also differences between the present and the previous study (Bai et al. 2021), which likely relate to the older age of the children in the present study. Children aged five to six years generated more ideas and these ideas were also more original, while the peak in originality was reached later in the sequence of all generated uses than in four-year-olds. A further finding of the current study, replicating our previous work (Bai et al. 2021), was that the domain-specific DT process mental operations was a positive predictor of originality, although this effect was somewhat weaker than it was in the previous study (for the five- to six-year-olds, the effect size expressed as Odds Ratio was *OR* = 1.38; while for the four-year-olds this was *OR* = 1.68). We proposed, as an interpretation, that performing mental operations on the stimulus is executive in nature, given that during this type of process, one would have to hold an image of the object in mind while manipulating it, inhibit the perceptual salience of the object in its usual whole integrated form, and shift attentional focus from global, holistic to local, singled-out features of the object in order to operate on these features. In particular, the mental process of singling-out properties or parts of whole objects and subsequently (re)combining these properties or parts into a new whole is considered conducive for generating original ideas in several theoretical (Lockman 2000) and empirical studies (Bai et al. 2021; van Dijk et al. 2020; Forthmann et al. 2016; Gilhooly et al. 2007; Nusbaum and Silvia 2011). The present findings provide additional, though indirect, support for this interpretation.

The current study further explored how domain-specific processes (mental operations, memory retrieval) and domain-general executive processes (EFs) relate to the generation of original ideas and in particular to the serial order effect. We tested our hypotheses by examining the interactions of DT processes by use rank, DT processes by EFs, and EFs by use rank. 

First, we expected the process of mental operations to become an increasingly important predictor of originality with increasing rank of the uses mentioned for a particular stimulus object, due to a gradual shift from responses based on associative memory processes (comparatively less original) to responses based on controlled mental operations (comparatively original). There was indeed an interaction effect of mental operations and use rank in predicting the originality of uses. However, contrary to our expectation, the interaction effect was negative. Based on the plot in Figure 1 (the green curves), the effect of mental operations on originality was already present in the initial stage of generating novel uses, remaining stable roughly until rank number six, but declining thereafter and turning even into a negative effect after rank 10 for the remaining 3% of all mentioned uses. In our previous study with the same children at age four years, based on a plot that modelled the occurrence probability of the process mental operations in relation to generated uses, the use of mental operations was virtually absent in the beginning of the sequence (Bai et al. 2021). A possible explanation is that the AUT was approached in a different way by the children in the current study. After successive testing with the AUT (also on two measurement occasions between four and five- to six years, not reported in this study) and due to being older and more accustomed to the structured context of the kindergarten, children may have understood the task demands better, complied more with the instruction of coming up with real novel uses, and therefore shifted almost immediately to the use of controlled executive processes, in particular mental operations, instead of initially relying on spontaneous memory-based associations.

To support this interpretation further, also the involvement of the DT process memory retrieval was different in the current compared to the previous study. Memory retrieval was considered to be mainly automatic and associative, and to rely on the automatic spreading of activation in episodic and semantic long-term memory (Gilhooly et al. 2007; Mednick 1962; Sowden et al. 2015), and, therefore, not expected to be a positive predictor of originality. The main effect of memory retrieval on originality, however, was positive and significant in the current study (*OR* = 1.13), though less strong than the effect of mental operations (*OR* = 1.38). In the previous study, there was no main effect of memory retrieval and, most importantly, memory retrieval was especially frequently referred to by the children explaining how they came up with an idea early in the sequence, right from the start, whereas mental operations were proportionally more frequently referred to at a later stage in the sequence of generated uses (Bai et al. 2021). Both the positive main effects of the DT processes mental operations and memory retrieval, and the timing of these effects underscore that children may have approached the AUT differently at age five to six years compared to when they were four years of age. Note that also in the study of Gilhooly et al. (2007) among university students, memory-based DT processes positively predicted originality. Thus, together, the findings of these studies seem to suggest that very young children (i.e., four-year-olds) may mostly rely on memory processes to generate relatively mundane ideas (e.g., “a basket is for carrying food, because I have seen that before”, as a participating four-year-old child explained; Bai et al. 2021), while older children and adults appear to be better able to use—potentially unique—memories to generate original ideas (e.g., “[use a toothbrush] to make a giraffe… I have seen that in films, and they use cardboard and sometimes also toothbrushes and other things, and then you make an insect…I had seen once that they used the toothbrush[es] as the neck and the legs”, as a child of the current study explained). Clearly, further longitudinal work is required to test this hypothesis. 

Finally, for both mental operations and memory retrieval, the interaction effect with use rank was negative and the plots in Figure 1 revealed a turning point for both processes between ranks 8 to 10 regarding the relation with originality, which turned negative. A possible explanation for this unexpected finding is that effectively applying mental operations and also memory retrieval requires effort, and children may have become tired after effortful thinking up about six or seven uses. If depletion indeed also played a role in using memory retrieval, this may further support the idea that also the use of this process differed from the previous study in that, instead of generating automatic, unfiltered responses from memory, additional control of the novelty of a generated use was applied by the children by inhibiting associative, yet less original responses. 

Second, we hypothesized that individual differences in EFs would interact with the DT process mental operations in the prediction of originality. However, none of the main effects of the EFs shifting, inhibition, and working memory on originality (all tested in separate models), nor the interaction effects of these EFs and mental operations were found to be significant, which is inconsistent with previous research involving mostly older children and adults. Although the association between inhibition and DT measures such as fluency and originality is inconclusive based on previous research, shifting and working memory have been rather consistently found to be positively related to DT measures in most studies (de Dreu et al. 2012; Kharkhurin and Wei 2015; Lee and Therriault 2013; Lu et al. 2017; Nusbaum and Silvia 2011; Yeh et al. 2015; Zabelina et al. 2019; Zabelina and Robinson 2010). Possibly measurement issues have played a role here. There were ceiling effects in the two inhibition tasks, indicating that these tasks may have been too easy for children in the current age range, although the tasks were selected for being age-appropriate. Further research is needed to investigate if and how EFs and DT are related at this age. 

Like the other domain-general EFs, selective attention had no main effect on originality, contrary to the expectation. However, a significant interaction effect of selective attention and use rank on originality was observed, that is, selective attention was found to moderate the serial order effect of originality. This moderation effect, plotted in Figure 1 (the orange curves), however, was negative and indicated that children with a lower level of selective attention produced more original responses over time than children with a higher level of selective attention already, from about rank five onwards. This result is in line with the findings of a number of recent studies on the role of inhibition and selective attention, showing an advantage for creativity and DT of low inhibition and low selective attention (van Dijk et al. 2020; Radel et al. 2015; Stolte et al. 2019; Zabelina 2018). A possible explanation is that lower selective attention facilitates perceiving a broader set of external stimuli (such as irrelevant objects in the test environment or additional test stimuli to promote novel combinations of stimulus features; cf. van Dijk et al. 2020) and promotes a broader spreading of activation to more remote and unrelated memory representations in semantic and episodic memory (Benedek et al. 2012b; Brown 1973; Mednick 1962), which would then lead to more original responses.

To summarize the findings, the present study provides tentative evidence for a complex interplay of different cognitive processes during DT, both controlled (the DT processes mental operations and memory retrieval) and less controlled (uncovered by the facilitating effect of low selective attention). The role of controlled processes early in the sequence of generating novel uses for a particular stimulus, as found in this study, seemed to reflect children’s compliance with the task demands, while the uncontrolled processes, facilitated by a broadened attentional scope, gradually replaced the controlled processes, possibly due to depletion of the latter, towards the end of the sequence.

### Strengths, Limitations, and Future Research

To the best of our knowledge, the present study is the first to systematically study how domain-general and domain-specific executive processes jointly operate during DT in young children with as yet limited formal schooling. Applying multilevel logistic regression analysis allowed us to study in a fine-grained way how DT upon a presented stimulus unfolds in real time and is moderated by both domain-general (selective attention) and domain-specific executive processes (mental operations, memory retrieval). In this way, the present study revealed a complex interplay of different cognitive processes underlying the generation of original responses in young children. A particular strength of the current approach was the multilevel modelling of both originality and DT processes. Detailing the processes of DT and creativity in future research may yield more conclusive evidence in particular regarding how individual differences in domain-general cognitive abilities such as EFs play out in the unfolding process of DT and creativity.

The present study suffered from a number of limitations. One important limitation is that the five- to six-year-olds in the current study had already been tested with the AUT three times at earlier measurement waves within the longitudinal project of which this study was part, although a different (equivalent) stimulus set was used at two of those earlier waves. As discussed above, the repeated assessments could have facilitated children’s understanding of the test instructions and thus, influenced how they approached the task. In this regard, caution is warranted when generalizing the results of the present study.

Another clear limitation is the small sample size relative to the complex statistical models, which may have reduced the power of the analyses. The power problem may have been enlarged by another limitation, the ceiling effects in some of the EF tasks that were apparently too easy for children of the current age range. However, the use of the complex models (such as M5 models shown in Table 6), consisting of effects that are inter-dependent, was necessary to test our hypotheses. Note that all models presented in the manuscript converged without problems despite their complexity. However, given the potential power issue, the fact that several expected effects were not found in the current study may not point to an absence of these effects but rather that these effects were small in the current study. It should be noted, as discussed in the Introduction, that the evidence regarding the relationships between EFs and DT (in particular regarding EFs and the serial order effect in DT) is not consistent (Beaty and Silvia 2012; Hass 2017; Wang et al. 2017). New research is recommended to test the current hypotheses again with larger samples, using EF tasks that are better able to capture the full ability range of children in the current age range. 

Another notable limitation relates to the measurement of the construct EF. In the current study, we treated inhibition, shifting, working memory, and selective attention as different executive functions and used separate tasks to measure them, following common practice (cf., Miyake et al. 2000). This approach allowed us to relate to studies with other—mostly adult—samples, which have reported differential associations between different EFs and DT (e.g., Wang et al. 2017). However, recent research suggests that EFs may be less differentiated in younger than in older children and adults (Karr et al. 2018). Perhaps more optimal modelling of EFs would have revealed different effects of EFs on DT in the current study. Future research is recommended to carefully select age-appropriate EF tasks and to examine their latent factor structure prior to the main analysis which is feasible when working with larger samples than the one in the current study (for more elaborate recommendations, see Mulder et al. 2014; van der Ven et al. 2012; Veer et al. 2017). 

Other limitations also deserve attention in future research. Regarding the AUT task, the test environment and the procedure of giving verbal prompts should be more standardized in order to reduce random noise (e.g., objects present in the test environment could affect DT performance) and experimenter-variance (e.g., experimenters had to decide when and how many prompts were given to children). In addition, for about half of all generated uses, children did not give an explanation or gave a non-codable explanation of their thinking processes. In future research, alternative methods should be applied in order to elucidate the thinking processes involved in DT more optimally, for example, by carrying out in-depth content analysis of children’s responses. Finally, the current study was conducted in a sample of children attending kindergarten classrooms within Dutch primary schools. Given the wide variety of care and education arrangements in early childhood across the world, replication of the findings of the current study in different educational contexts is clearly warranted.

## 5. Conclusions

The present study replicated the findings of a previous study and confirmed that there is a serial order effect in the originality of ideas generated by young children on the widely used divergent thinking task AUT. Both domain-general executive functions (selective attention) and domain-specific executive processes played a role in the real-time unfolding of original ideas, showing a complex interplay during DT. The processes of memory retrieval and applying mental operations on the stimulus was positively related to the early emergence of original ideas, while a lower level of selective attention facilitated original ideas in a later stage of idea generation. We could not confirm the expected role of the domain-general EFs shifting, inhibition and working memory, as none of these EFs predicted originality or moderated the serial order effect in originality. The current study underscores the value of a process approach in studying DT. Future research is needed to further elucidate the processes that play a role in the generation of original ideas from early childhood to adulthood. 

## Figures and Tables

**Table 1:** Definitions and Examples of Coded Divergent Thinking (DT) Processes in the Alternative Use Task (Bai et al. 2021).

DT Process Category	Definition	Examples (C = Child; T = Experimenter)
1. Retrieval or recall of prior knowledge or experience ( Memory retrieval)	There is clear evidence that the child refers to prior knowledge or prior experience while generating a use. The child may recall a specific memory of a real personal experience or a memory related to here—say, a story, film, or book that relates to the use. Or: The child gives an affirmative answer when asked by the experimenter if he or she had prior personal experience with the use (i.e., if he or she did/learned it before) or if the child knew about the use from others, stories, movies, or books.	1. C: “I always do that with my father.” 2a. T: “Have you done that before?” 2b. C: “Yes, I have done it once. “
2. Performing mental operations on the stimulus (Mental operations)	The child mentions or refers to a mental operation applied to the stimulus (e.g., disassembling, re-assembling, turning, distorting, folding, etc.) or the child proposes an (imagined) act of assembling, combining, or synthesizing the stimulus with other objects or materials, to obtain a functional change of the stimulus that enables the discovery of a use.	1. C: “If you take off these hairs (toothbrush) and then put such a brush on, and also paper on, then you can make a mouse. “ 2. C: “If you attach a lot of balloons on it (basket), which keeps floating, a lot a lot, then you can sit in there just like a hot air balloon. “

**Table 2:** Descriptive Statistics of Child-Level Measures Over Different Tasks.

Variables		*N*	*M*	*SD*	Skew.	Kurt.	Min.	Max.
**Divergent Thinking (DT) ability measures (mean scores per stimulus):**								
Fluency		101	4.21	1.79	1.58	2.96	1.00	11.00
Originality		101	2.13	1.29	1.93	5.43	0.09	7.90
**Frequency of DT processes (mean counts per stimulus):**								
Memory retrieval		101	1.80	1.05	1.35	2.90	0	6.00
Mental operations		101	0.50	0.69	2.59	7.49	0	3.67
**EF measures:**								
Inhibition	Go/NoGo: number of errors	100	3.61	2.82	1.93	8.06	0	19.00
	Animal stroop: proportion accuracy	100	.93	0.08	−4.27	26.49	.38	1.00
Shifting	DCCS: level achieved	96	2.34	0.50	0.41	−1.21	1	3.00
	Animal shifting: proportion accuracy	102	.91	0.09	−2.07	5.06	.50	1.00
Working memory	Word recall (backwards): proportion accuracy	98	.65	0.20	−0.001	−0.58	.17	1.00
Selective attention	Elephant test: average number of targets found	101	6.79	0.62	−0.82	0.92	4.75	8.00

*Note.* DCCS = The Dimensional Change Card Sort task.

**Table 3:** Correlations between Child-Level Measures over Different Tasks.

Variables	2	3	4	5	6	7	8	9	10	11
1. Fluency	.90 **	.052	.67 **	.44 **	.04	.03	*−.004*	−.04	−.06	.08
2. Originality		.15	.52 **	.59 **	−.05	.10	*.02*	.01	−.04	.11
3. Age			−.05	.06	−.08	.10	*−.06*	.12	.06	.09
4. Memory retrieval	−.27 **			.19 ^+^	.11	.04	*−.09*	−.08	.03	.11
5. Mental operations	.51 ***	−.16			−.15	.11	*.10*	.10	.01	−.05
6. Go/NoGo: number of errors						−.21 *	*−.07*	−.17	−.10	−.02
7. Animal stroop: proportion accuracy							*.11*	.38 **	.07	.07
8. DCCS: level achieved								.21 *	.19 *	*−.06*
9. Animal shifting: proportion accuracy									.16	.21 *
10. Word recall (backwards): proportion accuracy										.14
11. Elephant task: average number of targets found										

*Note.* DCCS = The Dimensional Change Card Sort task. Correlations below the diagonal are partial correlations corrected for fluency. Correlations in italics are nonparametric correlations and estimated with Kendall’s tau_b, as DCCS consisted of ordinal data. ^+^ *p* < .10. * *p* < .05. ** *p* < .01. *** *p* < .001.

**Table 4:** Number of Uses and Counts of Divergent Thinking Processes by Use Rank.

Use Rank	Number of Uses	Memory Retrieval	Mental Operations
1	598	212	12
2	497	260	54
3	420	206	66
4	334	151	61
5	232	93	32
6	147	54	25
7	96	41	19
8	64	21	9
9	45	17	6
10	35	12	4
11	24	7	3
12	19	7	4
13	12	4	2
14	8	1	2
15	5	1	1
16	3	0	1
17	3	0	1
18	3	1	0
19	2	2	0
20	1	1	0
21	1	0	0
22	1	0	0
Number of uses	2550	1091	302
Mean use rank	3.63	3.47	4.48
Number of participants	101	99	75

**Table 5:** Multilevel Logistic Regression Analyses With the Originality of Uses as the Outcome Variable, Examining the Linear and Quadratic Effects of Use Rank (i.e., the Serial Order Effect) and the Effects of Divergent Thinking (DT) Processes in Predicting the Logit Probability of the Originality of Uses (N = 101, with 606 units at the object level and 2550 units at the use level).

Models	Intercept-Model		Serial Order Effect		DT Process: Memory Retrieval (MR)				DT Process: Mental Operations (MO)			
	M1		M2		M3–MR		M4–MR		M3–MO		M4–MO	
**Fixed part**	Coeff. (s.e.)	*OR*	Coeff. (s.e.)	*OR*	Coeff. (s.e.)	*OR*	Coeff. (s.e.)	*OR*	Coeff. (s.e.)	*OR*	Coeff. (s.e.)	*OR*
Intercept	−5.11 (0.03) ***	0.006	−5.05 (0.03) ***	0.006	−5.06 (0.03) ***	0.006	−5.05 (0.03) ***	0.006	−5.06 (0.03) ***	0.006	−5.06 (0.03) ***	0.006
Linear: Use rank			0.15 (0.01) ***	1.17	0.16 (0.01) ***	1.17	0.16 (0.01) ***	1.17	0.15 (0.01) ***	1.16	0.15 (0.01) ***	1.16
Quadratic: Use rank			-0.014 (0.002) ***	0.99	−0.013 (0.002) ***	0.99	−0.014 (0.002) ***	0.99	−0.013 (0.002) ***	0.99	−0.012 (0.002) ***	0.99
DT process					0.12 (0.05) *	1.13	0.14 (0.05) **	1.15	0.32 (0.07) ***	1.38	0.39 (0.07) **	1.47
DT process × Use rank							−0.033 (0.02) ^+^	0.97			−0.056 (0.03) *	0.95
**Random part: Variance (s.e.)**												
σ^2^_object level_	0.00 (0.0002)		0.00 (0.0002)		0.00 (0.0002)		0.00 (0.0002)		0.00 (0.0002)		0.00 (0.0001)	
σ^2^_child level_	0.00 (0.0002)		0.00 (0.0001)		0.00 (0.0001)		0.00 (0.0001)		0.00 (0.0001)		0.00 (0.0001)	
Model deviance (*df*)	4386.80 (3)		4241.79 (5)		4235.97 (6)		4232.68 (7)		4220.82 (6)		4215.91 (7)	
Δdeviance (vs. a model, Δ*df*)			145.01 (vs. M1, 2) ***		5.82 (vs. M2, 1) *		3.29 (vs. M3–MR, 1) ^+^		20.97 (vs. M2, 1) ***		4.91 (vs. M3–MO, 1) *	
Scale ^a^	0.56		0.50		0.50		0.50		0.49		0.49	

*Note.* Deviance = −2 × log-likelihood. OR = Odds ratio. ^a^ The estimations of “scale” indicates variance dispersion. In the current study, the scales are all smaller than 1, indicating that the observed variance of the outcome measure originality is smaller than the theoretical variance (i.e., π^2^/3) of a binomial distribution. ^+^ *p* < .10. * *p* < .05. ** *p* < .01. *** *p* < .001.

**Table 6:** Multilevel Logistic Regression Analyses With the Originality of Uses as the Outcome Variable, Examining Interaction Effects of EFs, DT Processes, and Use Rank in Predicting the Logit Probability of the Originality of Uses.

Models	M5: Memory Retrieval (M5–MR)								M5: Mental Operations (M5–MO)							
	Inhibition		Shifting		Working Memory		Selective Attention		Inhibition		Shifting		Working Memory		Selective Attention	
**Fixed part**	Coeff. (s.e.)	*OR*	Coeff. (s.e.)	*OR*	Coeff. (s.e.)	*OR*	Coeff. (s.e.)	*OR*	Coeff. (s.e.)	*OR*	Coeff. (s.e.)	*OR*	Coeff. (s.e.)	*OR*	Coeff. (s.e.)	*OR*
Intercept	−5.06 (0.03) ***	0.006	−5.06 (0.03) ***	0.006	−5.06 (0.03) ***	0.006	−5.05 (0.03) ***	0.006	−5.07 (0.03) ***	0.006	−5.06 (0.03) ***	0.006	−5.06 (0.03) ***	0.006	−5.06 (0.03) ***	0.006
Linear: Use rank	0.15 (0.01) ***	1.17	0.15 (0.01) ***	1.17	0.15 (0.01) ***	1.17	0.16 (0.01) ***	1.17	0.15 (0.01) ***	1.16	0.15 (0.01) ***	1.16	0.15 (0.01) ***	1.16	0.15 (0.01) ***	1.16
Quadratic: Use rank	−0.015 (0.002) ***	0.99	−0.014 (0.002) ***	0.99	−0.014 (0.002) ***	0.99	−0.015 (0.002) ***	0.99	−0.013 (0.002) ***	0.99	−0.013 (0.002) ***	0.99	−0.013 (0.002) ***	0.99	−0.013 (0.002) ***	0.99
DT process	0.15 (0.05) **	1.16	0.17 (0.05) **	1.18	0.15 (0.05) **	1.16	0.15 (0.05) **	1.16	0.38 (0.08) ***	1.46	0.38 (0.08) ***	1.46	0.38 (0.07) ***	1.47	0.38 (0.07) ***	1.47
DT process × Use rank	−0.031 (0.02)	0.97	−0.035 (0.02) ^+^	0.97	−0.032 (0.02)	0.97	0.034 (0.02) ^+^	0.97	−0.060 (0.03) *	0.94	−0.056 (0.03) *	0.95	−0.055 (0.03) *	0.95	−0.060 (0.03) *	0.94
EF	0.031 (0.04)	1.03	0.040 (0.03)	1.04	−0.014 (0.03)	0.99	0.015 (0.03)	1.01	0.011 (0.04)	1.01	0.023 (0.03)	1.02	−0.013 (0.03)	0.99	0.024 (0.03)	1.02
Use rank × EF	0.019 (0.01)	1.02	0.009 (0.01)	1.01	0.003 (0.01)	1.00	−0.017 (0.00) ^+^	0.98	0.019 (0.01)	1.02	0.009 (0.01)	1.01	0.001 (0.01)	1.00	−0.017 (0.01) *	0.98
DT process × EF	−0.039 (0.07)	0.96	−0.008 (0.07)	0.99	−0.007 (0.05)	0.99	−0.010 (0.05)	0.99	0.004 (0.11)	1.00	−0.013 (0.09)	0.99	−0.029 (0.08)	0.97	−0.026 (0.07)	0.97
**Random part: Variance (s.e.)**																
σ^2^_object level_	0.00 (0.0002)		0.00 (0.0002)		0.00 (0.0002)		0.00 (0.0002)		0.00 (0.0001)		0.00 (0.0001)		0.00 (0.0001)		0.00 (0.0001)	
σ^2^_child level_	0.0002 (0.0007)		0.0002 (0.0007)		0.0002 (0.0007)		0.0001 (0.0005)		0.0001 (0.0005)		0.0001 (0.0005)		0.0001 (0.0005)		0.0001 (0.0004)	
σ^2^_use rank at child level_	0.0003 (0.0004)		0.0003 (0.0004)		0.0002 (0.0004)		0.0001 (0.0003)		0.0003 (0.0004)		0.0003 (0.0004)		0.0002 (0.0004)		0.0001 (0.0003)	
σ^2^_DT process at child level_	0.00 (0.0004)		0.00 (0.0004)		0.00 (0.0005)		0.00 (0.0005)		0.0006 (0.0024)		0.0008 (0.0029)		0.0006 (0.0025)		0.0003 (0.0022)	
Model deviance (*df*)	4011.66 (15)		3964.13 (15)		4120.28 (15)		4165.49 (15)		3997.16 (15)		3952.87 (15)		4104.94 (15)		4148.92 (15)	
**Scale ^a^**	0.50		0.50		0.50		0.50		0.49		0.49		0.49		0.49	

*Note.* Deviance = −2 × log-likelihood. OR = Odds ratio. *N* is 97, 95, 97, and 100 for models including inhibition, shifting, working memory, and selective attention respectively. ^a^ The estimations of “scale” indicates variance dispersion. In the current study, the scales are all smaller than 1, indicating that the observed variance of the outcome measure originality is smaller than the theoretical variance (i.e., π^2^/3) of a binomial distribution. ^+^ *p* < .10. * *p* < .05. ** *p* < .01. *** *p* < .001.

## Data Availability

The data presented in this study are available on request from the corresponding author. The data will be made publicly available when the research project of which this study is part is finished.

## Appendix A

**Table A1:** Main Types of Actions Used for Classifying Uses and Scoring Their Originality.

No.	Lunch box	Tire	Pencil	Umbrella	Shovel	Toothbrush
1	To put food in/as a food box	For cars/to ride with	For drawing	For (/to protect you from) rain(ing)	To shovel/dig	To clean teeth
2	To put something in/as a storage box	For making a swing	For writing	As a boat	To beat something or someone/to break something	To clean something/as a cleaning brush
3	To put insect or animal in/as an insect box	To play with (unspecified)	To erase something (use the attached eraser)	As a sunshade/against the sun	To put something on or in	To clean someone
4	For cut-and-glue activity/for artwork/to make something (unspecified)	For sitting or lying on	For coloring	To put something in	For fighting/as a sword	For painting or drawing
5	To put water or drink in	To sit in and roll	To poke something/to break something	For flying (with it)	To build or make something with sand	To sweep something/as a broom
6	As a garbage bin/to put dirty things in	To roll with (a sort of game)	For scratching	For beating something	To pick up or lift something	To brush hair/as a comb
7	For catching something	As a hula hoop	For cut-and-glue activity/to make something (unspecified)	For fighting/as a sword	For planting	For cut-and-glue activity/to make something (unspecified)
8	For taking something with you	For jumping in	For beating something	For protecting yourself/as a shield	For building or construction work	To play with (unspecified)
9	To write/draw something on	For making a small house	To draw something for cut-and-glue activity	To write/draw something on	For looking for something (usually in sand)	For fighting/as a sword
10	For making a boat	To put something in and roll	For painting	As a merry-go-round/carousel	To chop or hack something	For pretending to brush your teeth.

Note. Here we only present action types that were mentioned by at least five children (5% of the 101 children).

## Appendix B

**Table A2:** Inter-Coder Reliability Overview and Kappa’s for Divergent Thinking (DT) Processes (399 episodes).

DT Processes	Frequency of Coded Episodes by the Two Coders				Kappa’s
	Yes–Yes	Yes–No	No–Yes	No–No	
Memory retrieval	151	25	14	209	.80
Mental operations	34	21	5	339	.69

## Appendix C

**Table A3:** Missing Data of Different Tasks and Reasons for the Missing.

Tasks		Final Sample Size	Missing Data and the Reasons
Divergent thinking	Alternative Uses Task	101	1 child was not present on test dates;
Inhibition	Go/NoGo	100	1 child was simply hitting the key all the time; 1 child’s data file was overwritten by mistake.
	Animal Stroop	100	1 child was sick on test dates; 1 child’s test session was interrupted.
Shifting	Animal Shifting	102	No missing data for this task.
	Dimensional Change Card Sort	96	1 child’s recording form was lost; 5 children were not tested following the instructions from the manual (i.e., the reminders of rules were not given while a stimulus was presented to children).
Working memory	Word recall	98	4 children showed clear indications of not understanding the instructions (i.e., try to recall the words in reversed alphabet order)
Selective attention	Elephant search	101	1 child was sick on test dates.
